# Cognitive behavioral therapy for insomnia vs. standard cognitive behavioral therapy for sleep and circadian disturbances in treatment-resistant schizophrenia: study protocol for the randomized controlled trial (COSTS)

**DOI:** 10.1186/s13063-026-09482-0

**Published:** 2026-04-17

**Authors:** Jeppe Feigenberg Johansen, Mette Ødegaard Nielsen, Mette Kragh, Jimmi Nielsen

**Affiliations:** 1 Unit for Complex Schizophrenia, Capital Region Mental Health Services, Nordstjernevej 20, 2600 Glostrup, Denmark; 2 Faculty of Health and Medical Sciences, Department of Clinical Medicine, University of Copenhagen, Copenhagen, Denmark; 3 Department of Clinical Medicine, Department of Affective Disorders, Aarhus University, Palle Juul-Jensens Boulevard 175, Aarhus N, 8200 Denmark

**Keywords:** Schizophrenia, Psychosis, Treatment-resistance, Cognitive behavioral therapy, Insomnia, Sleep disturbances, Antipsychotics, Polysomnography, Randomized controlled clinical trial

## Abstract

**Background:**

A subset of patients with schizophrenia do not respond sufficiently to conventional antipsychotic treatment and often have a more complex clinical course, including high rates of sleep disturbances, which can contribute to further worsening of symptoms. However, sleep disturbances are often overlooked in clinical psychiatric settings, and non-pharmacological treatment options are not initiated. Cognitive behavioral therapy for insomnia (CBT-I) has been shown to effectively ameliorate sleep disturbances in schizophrenia but is yet to be assessed in treatment-resistant schizophrenia. In the present study, we aim to investigate the efficacy of CBT-I versus standard cognitive behavioral therapy (CBT), an active control intervention.

**Methods:**

Sixty patients diagnosed with treatment-resistant schizophrenia and comorbid sleep disturbance will be included in this randomized intervention study. Included patients will be randomized to 8–10 sessions of psychotherapy with either CBT-I (active intervention) or regular CBT (active control) following baseline. At baseline and 12-week follow-up, patients will be assessed with clinical interviews (Positive and Negative Syndrome Scale), self-reported measures (e.g., Insomnia Severity Index), and polysomnography. The 24-week follow-up will include the same assessments apart from polysomnography. The active intervention group will receive an individual course of treatment with CBT-I focused on the patients’ sleep patterns, while the active control group will receive an individual course of treatment with standard cognitive behavioral therapy (CBT) focused on patients’ psychopathology. It is hypothesized that while both groups will show improvements on central outcome measures, CBT-I will show greater improvements in sleep disturbances. Further, it is hypothesized that the improvement in sleep disturbances will correlate with an improvement in positive symptoms. Lastly, it is anticipated that the CBT-I group will show objective improvements in sleep architecture, such as sleep latency, wake after sleep onset, sleep efficiency, and total sleep time, compared to the CBT group.

**Discussion:**

Should CBT-I prove efficacious in improving sleep disturbances in treatment-resistant schizophrenia, it would provide an avenue for a cost-beneficial, short-term, and implementable non-pharmacological treatment of a severe comorbidity in complex schizophrenia patients. Potential issues pertaining to the completion of the study are discussed.

**Trial registration:**

ClinicalTrials.gov NCT06749444. Registered on December 27, 2024.

## Administrative information

Note: the numbers in curly brackets in this protocol refer to SPIRIT checklist item numbers. The order of the items has been modified to group similar items (see http://www.equator-network.org/reporting-guidelines/spirit-2013-statement-defining-standard-protocol-items-for-clinical-trials/).

**Table:** 

Title {1}	Cognitive behavioral therapy for insomnia vs. standard cognitive behavioral therapy for sleep and circadian disturbances in treatment-resistant schizophrenia: study protocol for the randomized controlled trial (COSTS)
Trial registration {2a and 2b}	ClinicalTrials.gov Identifier: NCT06749444
Protocol version {3}	Protocol version 1.5, 11th June 2025.
Funding {4}	DKK 2.243.184 from Independent Research Fund Denmark to conduct the present study under the call Thematic Research - Better Frameworks for Psychiatry (2023). The PhD-student and the nurse on the study will be paid with these funds. DKK 36.000 from Grosserer L. F. Foghts Fond for the external rating of PANSS. DKK 100.000 from Slagtermester Wørzner og hustru Inger Wørzners mindelegat til forskning i sindslidelser for the supervision of the therapist in the CBT-I group. DKK 200.000 from Aase og Ejnar Danielsens Fond for external rating of PSG. DKK 216.000 from Læge Sofus Carl Emil Friis og hustru Olga Doris Friis’ legat for the therapist conducting the CBT-intervention.
Author details {5a}	Jeppe Feigenberg Johansen - Unit for Complex Schizophrenia, Capital Region Mental Health Services, Nordstjernevej 20, 2600 Glostrup, Denmark & Faculty of Health and Medical Sciences, University of Copenhagen, e-mail: jeppe.feigenberg.johansen@regionh.dk, ORCID: 0009-0009-7609-2099 Mette Ødegaard Nielsen - Unit for Complex Schizophrenia, Capital Region Mental Health Services, Nordstjernevej 20, 2600 Glostrup, Denmark & Department of Clinical Medicine, University of Copenhagen, e-mail: mette.oedegaard.nielsen@regionh.dk, ORCID: 0000-0002-0780-7099 Mette Kragh - Department of Clinical Medicine, Department of Affective Disorders, Aarhus University, Palle Juul-Jensens Boulevard 175, 8200 Aarhus N, Denmark, e-mail: metkragh@rm.dk, ORCID: 0000-0001-6684-428X Jimmi Nielsen - Unit for Complex Schizophrenia, Capital Region Mental Health Services, Nordstjernevej 20, 2600 Glostrup, Denmark & Department of Clinical Medicine, University of Copenhagen, e-mail: jimmi.nielsen@regionh.dk, ORCID: 0000-0002-9868-7028
Name and contact information for the trial sponsor {5b}	Jimmi Nielsen, jimmi.nielsen@regionh.dk Unit for Complex Schizophrenia, Nordstjernevej 20, 2600 Glostrup, Denmark
Role of sponsor {5c}	The sponsor’s role in study is to contribute to the analysis and interpretation of data as well as writing and submitting papers for publication. However, the sponsor will not have ultimate authority over any of these activities. The funding bodies have had no influence on any aspect of the study.

## Introduction

### Background and rationale {6a}

Sleep is essential to physical and mental health [1] and is often disrupted in people with schizophrenia and can be observed leading up to first-episode psychosis [2], making sleep disturbances a comorbidity that permeates the course of illness. Up to 80% of schizophrenia patients experience insomnia-related symptoms, whereas 30–40% meet the criteria of clinical insomnia [3, 4]. Sleep disturbances are prevalent, but clinically often-overlooked comorbidity that is linked to more severe symptoms and worse clinical prognosis [2].

Schizophrenia can be a debilitating psychiatric disorder characterized by symptoms, such as cognitive impairments, hallucinations, delusions, inadequate affect, and lack of initiative and motivation [5]. Antipsychotic medication improves psychotic symptoms in most patients, but 30–40% do not experience sufficient, if any, symptom reduction despite treatment [6]. These patients are defined as treatment-resistant schizophrenia (TRS) and are characterized by complicated pharmacological treatment and resistant psychopathology [7], which is further exacerbated by the presence of a comorbid sleep disturbance.

Cognitive Behavioral Therapy (CBT) is the first-line treatment for a range of psychiatric illnesses and is generally an effective treatment option for most assessed conditions [8]. Traditional second-wave Beckian CBT generally results in small, but significant effects on symptoms in the treatment of schizophrenia, proving most effective in treating positive symptoms, while largely ineffective in treating negative symptoms [9]. Besides pharmacological treatment of sleep disturbances in patients with schizophrenia, the last number of years has brought a rise in popularity of interventions based on principles from CBT.

*Cognitive behavioral therapy for insomnia* (CBT-I), a niche branch of broader CBT, offers a promising treatment of sleep disturbances that appear comorbidly with another psychiatric illness that is efficacious in addition to being cost-effective compared to pharmacotherapy and easy to implement [10]. CBT-I is a multi-component treatment comprised mainly of sleep restriction therapy (SRT), stimulus control therapy (SCT), cognitive therapy, and sleep hygiene psychoeducation [11, 12]. These components address the restructuring of dysfunctional assumptions about sleep and insomnia, rumination and worry, behaviors pertaining to falling asleep and waking up, and elevation of daytime activity levels [2]. CBT-I has been efficacious in treating chronic insomnia alone [13] and comorbid insomnia in depression [14] and anxiety [15]. CBT-I has also been used to treat insomnia in schizophrenia [2, 16–20]. Furthermore, CBT-I has been shown to produce greater and more lasting effects than common pharmacotherapy for insomnia [21] However, to our knowledge, CBT-I has not previously been rigorously investigated in TRS where pharmaceutical interventions are insufficient.

Several studies have found a positive relationship between insomnia and paranoid thoughts [22, 23]. The causality remains largely unknown, but it is most likely a circular, self-perpetuating relationship. Insomnia, anxiety, and paranoia may exacerbate each other, since both insomnia and paranoia are closely linked with negative affect, such as depression, anxiety, and rumination [22, 23]. A recent meta- analysis showed that insomnia was present in 50% of patients with schizophrenia and that disruption of sleep was a significant predictor of both onset and persistence of psychotic symptoms [24]. This meta-analysis showed that most patients were motivated for treatment and the intervention studies reported large effect size improvements in sleep and more modest, although still significant improvements in psychotic symptoms [24].

One of the best validated self-report measures of sleep and circadian disorders is the *Insomnia Severity Index* (ISI). This instrument was developed by comparing ISI with data from sleep journals and polysomnography (PSG) and comparing self-report ISI with peer-report ISI obtained from clinicians and relatives. ISI was able to detect changes in the perceived level of insomnia and can be used as a screening tool as well as an outcome measure [25]. ISI has recently been validated in a Danish context in a somatic outpatient clinic, where ISI-DK showed good reliability and validity [26].

PSG is an objective measure of sleep disturbances and is considered the golden standard of sleep assessment. Sleep research has discovered disorder-specific and transdiagnostic patterns and mechanisms, which implies that sleep disturbances are present comorbidly in many mental disorders leading to impairment in several areas [27]. Insomnia is characterized as difficulties falling and staying asleep with repeated awakenings or sleep that is perceived as nonrestorative or of poor quality [28]. Abnormalities in the structure of sleep seem to be an inherent part of schizophrenia, which is supported by a recent meta-analysis of PSG studies, which found sleep continuity and depth to be fundamentally altered in schizophrenia [27].

Likewise, deficits in sleep spindles and increased sleep latency have been observed in schizophrenia patients compared to non-clinical populations [2]. Lower sleep efficiency, despite longer total time asleep, more nightly awakenings, and a significantly higher score on *Pittsburgh Sleep Quality Index* (PSQI), reflecting worse subjective sleep quality, has also been demonstrated [29].

Despite the well-documented association between schizophrenia and insomnia in the existing literature, there is currently a gap in knowledge regarding nonpharmacological treatment options for sleep disturbances in TRS. Patients suffering from TRS are a heterogeneous group burdened by symptoms and significant side effects from the pharmacological treatment. We aim to investigate whether CBT-I is a useful nonpharmacological treatment option for TRS patients with sleep disturbances.

### Objectives {7}

Using a randomized controlled design, the project aims to investigate whether CBT-I can significantly lessen the burden of the disrupted sleep in patients with TRS and by proxy lead to a decrease in psychotic symptoms and increased quality of life. We hypothesize:

1. CBT-I group will show greater improvements on *Insomnia Severity* Index (ISI) compared to active control group (primary hypothesis);
2. CBT-I group will show greater improvements on *Positive and Negative Syndrome Scale* (PANSS) compared to active control group;
3. The improvement in sleep disturbances will correlate with an improvement in positive symptoms as measured by PANSS;
4. CBT-I group will show objective improvement in sleep architecture on polysomnography (PSG) as measured by a decrease in sleep latency (SL) and wake after sleep onset (WASO), and an increase in sleep efficiency (SE) and total sleep time (TST) compared to the active control group.

### Trial design {8}

**Fig. 1:**
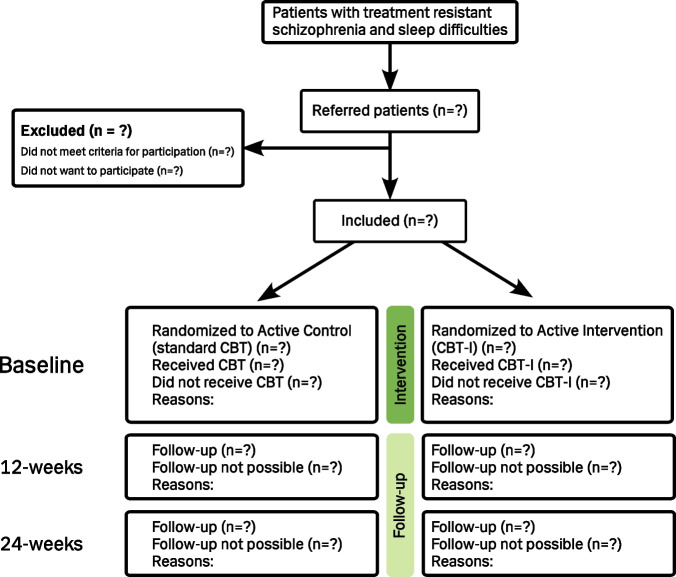
Flowchart of the COSTS trial

This is a 12-week non-blinded randomized controlled trial (RCT) with a superiority framework. Patients will be randomized 1:1 using a block randomization regimen with variable block sizes (2, 4, and 6), which is done to achieve equal sample size in different groups over time [30]. For a flowchart of the trial, see Fig. 1.

## Methods: participants, interventions, and outcomes

### Study setting {9}

The study will be conducted at the Unit for Complex Schizophrenia (UCS) at Mental Health Centre Glostrup, which is a university hospital located in Copenhagen, Denmark. UCS is a specialized psychiatric out-patient clinic, which has a steady referral of patients from the entire Capital Region for second opinions and complex treatments and has several active research projects on schizophrenia, including TRS.

### Eligibility criteria {10}

Patients with schizophrenia or other non-organic, chronic psychoses with a history of treatment-resistance can be included. In this project, we operationalize TRS as patients having positive symptoms that negatively influence their everyday function while receiving adequate psychopharmacological treatment. In addition, most of these patients will fulfill the strict definition of treatment-resistance (TRRIP-guidelines) [31], which states that participants have tried at least two antipsychotic drugs in sufficient dosage (equivalent to ≥ 600 mg chlorpromazine or half the dose of the maximum licensed dose) for sufficient time (≥ 6 weeks) without sufficient improvement in symptoms (moderate level of residual symptoms). Sleep disturbances are defined as sleeping difficulties pertaining to falling and staying asleep lasting ≥ 3 months [32] and a score of ≥ 15 on ISI [20]. Patients referred to UCS and fulfilling inclusion criteria will be offered to participate.

- Inclusion criteria:
  1. Diagnosed with ICD-10 schizophrenia (F20.x), chronic paranoid psychoses (F22), schizoaffective psychoses (F25.x), or other non-organic psychoses (F28–29)
  2. Ongoing positive symptoms equivalent to a score of at least 4 on PANSS positive item 1, 2, or 3 despite antipsychotic treatment at a sufficient dose
  3. Sleep disturbance (duration ≥ 3 months and ISI score ≥ 15)
  4. Stable pharmacological treatment for ≥ 1 month
  5. Age 18–64 years
  6. Legally competent
- Exclusion criteria:
  1. Admission to a psychiatric ward within the last 6 months that lasted > 1 week OR resulted in significant changes to the pharmacological treatment
  2. Substance abuse to a degree that could interfere with adherence to and compliance with intervention
  3. Diagnosis of sleep apnea and usage of CPAP

### Who will take informed consent? {26a}

Prior to obtaining informed consent, the participant will be thoroughly informed about the research project and their rights as research participants in both verbal and written form, including information about randomization, the different interventions, and pre- and post-intervention assessments. The patient will also be informed about the intent to videotape one therapy session to ensure treatment fidelity as well as the baseline and follow-up assessment of psychopathology. After receiving the information, the patient will have a relevant consideration period of at least 24 h before the consent is obtained.

### Additional consent provisions for collection and use of participant data and biological specimens {26b}

Not applicable, no ancillary studies planned.

## Interventions

### Explanation for the choice of comparators {6b}

To test the efficacy of CBT-I, we compare it to regular CBT with a focus on patients’ psychopathology. CBT in general is an evidence-based treatment for treating several types of symptoms in a long range of psychiatric illnesses [8], including schizophrenia [9, 33–35]. In Denmark, CBT is recommended as a first choice of psychotherapeutic intervention for schizophrenia by the Danish Health Authority [36]. No significant difference in RCTs has been found between CBT and other psychotherapies in patients with schizophrenia; however, patients receiving CBT were more satisfied with the intervention, as measured by adherence to treatment [37]. There is generally a lack of evidence of efficacy for other psychotherapeutic interventions in schizophrenia, making CBT the most obvious choice of comparison for CBT-I. CBT-I has also shown to be effective in treating sleep disturbances in a schizophrenia population [2, 16–18] as well as in other psychiatric illnesses [14, 15]. CBT-I is the first-line intervention for insomnia issues across conditions. Therefore, it seems appropriate to compare CBT-I to CBT.

In pharmacological research, the preferred option for trials with a superiority design is to use active control in lieu of placebo control [38]. Meanwhile, authors of a recent review agree that all trial designs are relevant to psychiatric research [39].

Active control groups are often used in RCTs in mental disorders research, and a recent meta-analysis found that groups receiving active control treatment showed a small improvement over those receiving placebo treatment, although this was not found in the trials on schizophrenia included in the meta-analysis [40]. In psychotherapy research, the evidence for active control over placebo control is murky. Expectation effects in psychological interventions are only partly eliminated by an active control group [41]. However, we address expectancy and placebo effects by changing only the focus of the intervention in each group, while using the same therapeutic techniques. Furthermore, by offering two evidence-based interventions and presenting both as such, the expectancy effects are mitigated.

### Intervention description {11a}

Included patients will be randomized 1:1 to CBT-I with a specific focus on sleep or regular CBT with a specific focus on patients’ psychopathology and related difficulties. The interventions are delivered by two different psychologists with similar experience in the field.

Patients randomized to the CBT-I arm will receive a manualized intervention, specifically adapted for individuals with severe mental illness and sleep disturbances. The intervention is delivered individually over 8–10 sessions at UCS, scheduled approximately once per week across a 12-week period. The duration of sessions varies according to the treatment phase:

- Session 1 (Initial assessment): 60–120 min
- Session 2 (Treatment initiation): 60–120 min
- Sessions 3–8 (Titration and relapse prevention): 45–60 min each

The extended duration of the first two sessions is necessary to allow for comprehensive assessment, individualized treatment planning, and thorough patient education.

The treatment protocol follows a Danish CBT-I manual developed for the COSTS project, which follows the general structure of previous manuals [42]. The first session includes a comprehensive assessment of sleep problems, confirmation of inclusion criteria, and introduction to the sleep diary, which patients are instructed to complete daily throughout the intervention. The sleep diary is used both for baseline assessment (between the first and second session) and as an ongoing tool to guide individualized treatment adjustments.

Core components of the intervention include:

- SRT: Based on the patient’s average TST from the sleep diary, an initial sleep window is set, with a minimum time in bed (TIB) of 5 h. Bedtime and rise time are fixed to strengthen circadian rhythms. Each week, the sleep window is titrated according to SE: if SE ≥ 85%, TIB is increased by 15 min; if SE is 80–84%, TIB is unchanged; if SE ≤ 79%, TIB is reduced by 15 min. Napping is discouraged throughout the intervention.
- SCT: Patients are instructed to go to bed only when sleepy, use the bed only for sleep and intimacy, leave the bed if unable to sleep after approximately 20 min, and maintain a consistent wake time regardless of sleep duration.
- Cognitive restructuring: The intervention targets unhelpful beliefs and worries about sleep, using cognitive techniques to challenge and modify maladaptive thoughts.
- Sleep hygiene and psychoeducation: Patients receive education on factors affecting sleep (e.g., caffeine, light, exercise), and are encouraged to optimize their sleep environment and routines.
- Relapse prevention: The final sessions focus on consolidating gains and developing strategies to prevent relapse.

The therapist follows a structured session plan, and adherence to the manual is monitored through continuous supervision by a senior clinical psychologist. The intervention is designed to be reproducible and evidence-based, following current recommendations for CBT-I [43].

The regular CBT intervention is delivered in a format identical to CBT-I. The structure, frequency, and duration of sessions are matched across both arms.

The primary difference is the therapeutic focus: regular CBT targets the participant’s psychopathology and related functional challenges, rather than sleep. Core components include psychoeducation, cognitive restructuring, behavioral experiments, coping skills training, and relapse prevention, with content individualized according to the participant’s needs.

Participants in the regular CBT arm complete a symptom log analogous to the sleep diary used in CBT-I, focusing on relevant psychiatric symptoms and coping behaviors. This log is reviewed in each session and guides the therapeutic process. This approach ensures both groups receive equivalent structure and intensity, with the main distinction being the content and focus of the intervention.

### Criteria for discontinuing or modifying allocated interventions {11b}

The psychotherapeutic intervention in both groups will be adjusted continuously for each trial participant given the individual nature. In the unlikely event that either intervention should result in a worsening of the psychotic symptoms, both the participant and the clinician providing the intervention can decide to discontinue the intervention, ideally in a way resembling shared decision-making.

Furthermore, 2 untimely cancelations or failures to appear in a row are considered non-compliance with the treatment, and the participant is excluded from the study.

Lastly, a change in a participant’s psychopathology which results in either substantial changes in antipsychotic medication or admittance lasting ≥ 7 days will result in exclusion from the study as per the exclusion criteria.

### Strategies to improve adherence to interventions {11c}

Improving treatment adherence from participants is a built-in feature of psychotherapy. There are two types of adherence in this project: (1) that participants show up for their appointments and (2) that they adhere to the prescriptions from the therapist in between sessions. While both types are important, the following paragraph is focused on the second type of adherence. During the study, patients will report a written overview of sleep and psychopathology symptoms (depending on assigned group). Therefore, any lack of or change in the level of adherence to treatment will be easily detected, and the therapist will be readily able to address these issues in the therapy session.

Measures to improve protocol adherence from the therapists will also be undertaken: to ensure treatment fidelity, 1 session with each patient will be recorded and assessed by an impartial therapist/supervisor. Furthermore, the filmed therapy session is used to provide supervision to the therapist in the active intervention group.

### Relevant concomitant care permitted or prohibited during the trial {11d}

Patients will continue their regular psychopharmacological treatment and attend visits with their regular care team at the referring outpatient clinic. Substantial changes in antipsychotic medication or hospital admittance lasting ≥ 7 days will result in exclusion from the study.

### Provisions for post-trial care {30}

Not applicable, no provisions for ancillary and post-trial care.

### Outcomes {12}

The primary outcome is improvement of the individual ISI score after 12 weeks. Secondary outcome measures are ISI score after 24 weeks and changes in PANSS (both total and subscale scores) [44], Global Assessment of Functioning (GAF) [45], WHO Well-Being Index (WHO-5) [46], Functioning Assessment Short Test (FAST) [47], Personal and Social Performance Scale (PSP) [48], the Process of Recovery Questionnaire (QPR) [49], the Treatment Expectation Questionnaire (TEX-Q) [50] and PSG. All outcomes are measured at baseline, after 12 weeks, and at 24 weeks, except for PSG, which is only measured at baseline and after 12 weeks.

### Participant timeline {13}

**Fig. 2:**
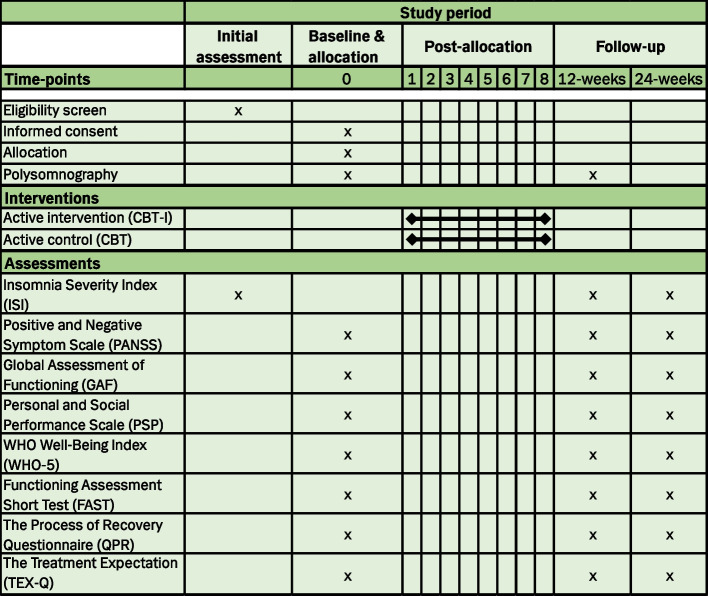
Overview of visits, assessments, and interventions

The inclusion period will be November 2024–December 2026. Patients included in the study will go through baseline assessments of approximately 3 h, including obtaining informed consent, randomization to intervention, and attachment of PSG equipment. Within two weeks of signing informed consent, the participant will be summoned for the first psychotherapy session in a course of 8–10 individual sessions. The sessions will take place over a period of approximately 12 weeks. Within two weeks of the final psychotherapy session, participants will be summoned for 12-week follow-up assessments, which are identical to the baseline assessments. 12 weeks later, participants will be summoned for a 24-week follow-up, which includes the same battery apart from the PSG. See Fig. 2 for an overview of visits, assessments, and interventions.

### Sample size {14}

To reach a power of 80% (*α* = 0.05, *β* = 0.2) for detecting a between-groups effect of CBT-I on ISI of 4, follow-up data on 24 patients in each group is needed. Since an attrition rate of around 25% is expected, the aim will be to include a total of 60 patients. For the power calculation, means and standard deviations from a previous study were used [20]. However, the between-groups effect is expected to be less since we are using two active interventions.

### Recruitment {15}

Leading up to and during the recruitment period, UCS will promote participation in the study internally in the Capital Region Mental Health Services, primarily through posters in the waiting areas of outpatient clinics and short lectures about the project to relevant clinicians. Further, complex schizophrenia patients referred for second opinion at UCS will be offered additional information on participation if they fit the target group.

## Assignment of interventions: allocation

### Sequence generation {16a}

The allocation sequence used is computer-generated using an online allocation tool (https://www.sealedenvelope.com/). This allows for randomization to be done with variable block sizes (2, 4, and 6) to achieve equal sample size in the two different groups over time.

### Concealment mechanism {16b}

The randomization process is concealed using opaque, sealed, and sequentially numbered envelopes. This concealment mechanism is done without the participation of those responsible for enrolling patients. Further, the envelopes are stored in a location unbeknownst to those enrolling patients. Patients will be given the next envelope in the sequence, which contains the intervention they are assigned to. The allocation process will take place in a separate room without the participation of those in charge of enrolling patients.

### Implementation {16c}

The allocation sequence is generated using the above online tool by an academic employee at UCS, who is not otherwise an active part of the study, apart from aiding with creating figures and tables for the article. The enrollment of participants is done by one psychologist (JFJ) and one nurse, who will manage the process from the first contact until assigning of intervention. The same academic employee will manage the process of assigning participants to the interventions.

## Assignment of interventions: blinding

### Who will be blinded {17a}

Most of the study will be unblinded. However, to ensure valid and unblinded rating of psychopathology, the PANSS interviews will be videotaped and rated by external raters.

### Procedure for unblinding if needed {17b}

Not applicable, no blinding planned for the intervention of the study.

## Data collection and management

### Plans for assessment and collection of outcomes {18a}

#### Assessment and collection of data

The following instruments will be implemented in the study:

- ISI is a brief seven-item questionnaire designed to measure perceived as well as changes in levels of insomnia, which has been thoroughly validated [25]. It includes items pertaining to nighttime impairment, such as trouble getting to sleep, staying asleep, and early morning awakenings, and daytime impairment, such as satisfaction, daily functioning, noticeability, and worries related to sleep patterns. Internal reliability, as measured by Cronbach’s coefficient alpha, ranged from 0.76 to 0.78 at baseline and follow-up evaluation. Concurrent validity, as measured by Pearson’s *r* coefficient, ranged from 0.07 to 0.91 across several information sources at baseline and follow-up. These results generally show a significant correspondence between patients’ self-perceived insomnia severity and that obtained from significant others and clinicians [25].
- PANNS is a semi-structured interview based on 30 items developed to assess the positive, negative, and general psychopathological symptoms of patients suffering from schizophrenia [44]. It has been extensively used in both clinical and research settings to measure and monitor the development of symptoms in schizophrenia. Internal reliability, as measured by Cronbach’s coefficient alpha, was 0.73, 0.83, and 0.79 for the Positive Scale, Negative Scale, and General Psychopathology Scale, respectively. Criterion-related validity (composed of discriminant and convergent validity), as measured by Pearson’s *r* coefficient, generally showed that the subscales of PANSS tend to correlate positively with related constructs and negatively with unrelated constructs. An overview of correlations can be seen in Kay et al. [44].
- GAF is a transdiagnostic rating instrument developed for objectively assessing the severity of psychiatric illness as it relates to the level of psychological, social, and occupational functioning [45]. This is rated in a single score ranging from 0 to 100 in 10-point intervals, which makes GAF an easy and quick tool to assess the level of functioning. GAF has shown high inter-rater reliability of 0.88 [48].
- PSP is a semi-structured interview developed to assess functioning in four separate dimensions for patients suffering from schizophrenia [48]. The four dimensions are socially useful activities, personal and social relationships, self-care, and disturbing and aggressive behaviors. This makes PSP a more in-depth instrument for assessing the level of functioning, which is an important variable in schizophrenia research. Inter-rater reliability for this instrument ranged from 0.68 to 0.90 across different occupations, professional backgrounds, and numbers of years working in mental health care [48].
- FAST is a 24-item instrument administered as a short interview designed to assess challenges in daily functioning in psychiatric patients, which is centered around six domains: autonomy, occupational functioning, cognitive functioning, financial issues, interpersonal relationships, and leisure time [47]. FAST has been demonstrated to have excellent internal consistency (Cronbach’s alpha = 0.89) and concurrent validity through a moderate positive correlation with the Brief Psychiatric Rating Scale and a strong negative correlation with the GAF scale [51].
- WHO-5 is a brief 5-item self-report measure used to assess levels of subjective psychological well-being, which is one of the most widely used questionnaires in this area [46]. Across several samples, WHO-5 has been shown to have excellent internal consistency with Cronbach’s alpha ranging from 0.83 to 0.95 [52]. Furthermore, WHO-5 has robust enough validity to warrant use as a well-being rating scale in clinical trials [46].
- QPR is an instrument used to assess the concept of recovery in psychiatric patients recovering from psychosis, which consists of two subscales, namely intrapersonal and interpersonal [49]. The revised version used in the present study is a 15-item instrument, which has been shown to have excellent internal consistency at a Cronbach’s alpha of 0.89 and a convergent validity at a Pearson’s *r* of 0.73 [53].
- TEX-Q is a 15-item generic instrument to assess treatment expectations in a multidimensional manner [50]. The overall scale has shown good internal consistency at Cronbach’s alpha = 0.79 with the subscales ranging from 0.71 to 0.91, while the test-retest reliability (as measured by Pearson’s *r*) was high for the overall scale (Pearson’s *r* = 0.76, *p* < 0.001) and ranging from *r *= 0.60 to 0.84, *p* < 0.001 for the subscales, except for the process subscale (*r* = 0.39, *p* = 0.026) [50].
- PSG is a comprehensive overnight sleep study that records multiple physiological parameters, including brain activity, eye movements, respiratory effort, airflow, and oxygen saturation [27, 54]. It measures sleep stages, breathing patterns, and limb movements to diagnose and evaluate sleep disorders such as sleep apnea, narcolepsy, and parasomnias [55]. In research, PSG is the gold standard for assessing sleep architecture and treatment outcomes. PSG demonstrates high validity and test-retest reliability for key sleep parameters, especially in controlled laboratory settings [56].

For PANSS, PSP and GAF, ongoing training of assessors is already implemented through co-rating at a weekly to monthly frequency.

An overview of the data collection plan at baseline and follow-up is provided in Fig. 2.

### Plans to promote participant retention and complete follow-up {18b}

Longitudinal follow-up data are important in clinical trials [57, 58]. Generally, issues with the collection of follow-up data can be summed up in the following categories: (1) unable to contact, (2) unable to complete, (3) refusing, and (4) unable to locate [57]. The loss of follow-up data due to subject attrition is a frequent cause of loss of statistical power or increased risk of bias, which minimizes the generalizability and validity of a clinical trial [57, 59]. Consequently, a robust plan for obtaining follow-up data is important. At the initial assessment, we focus on building rapport and positive interactions between participants and clinicians. This fosters participants’ commitment to both the project and staff [57]. We will build rapport through genuine interest in participants’ issues, history, and potential questions regarding project participation. Prospective participants are informed about the project’s goals, the importance of completing participation, and the personal benefits of participation. These strategies may raise the level of adherence throughout our study [59]. Obtaining accurate personal and demographic data is important in locating and contacting participants during the participation period. Furthermore, at the initial assessment, we will also schedule approximate dates for gathering follow-up data. Having scheduled appointments should increase the likelihood that participants will attend both follow-up sessions. Likewise, after participants have been assigned to a treatment group, the therapist in each group will send written reminders the day before each scheduled therapy session. A strong and continuous focus on overcoming these barriers should maximize the number of participants that complete the follow-up assessments.

### Data management {19}

Study data is collected by the research staff and managed using REDCap electronic data capture tools hosted at the Capital Region Mental Health Services. PSG data will be electronically stored on a secure server provided by the Capital Region Mental Health Services, Denmark.

### Confidentiality {27}

The project will comply with the data protection regulation and data protection act (Databeskyttelseslov- og forordning). The project has been approved by the Danish Data Protection Agency under the common Capital Region Mental Health Services’ umbrella application. All data on a secured data-drive (REDCap). REDCap is a secure, web-based software platform designed to support data capture for research studies [60]. Data extracted for analyses will be handled as confidential data and pseudo-anonymized using project letters and ID numbers, which will be kept at closed, secured, and monitored data-drives. PSG data will likewise be kept in pseudo-anonymized form in a secured data folder. Only anonymized data will be published.

The data will be stored in REDCap until 31 December 2039. Subsequently, the data will be archived in fully anonymized form.

### Plans for collection, laboratory evaluation and storage of biological specimens for genetic or molecular analysis in this trial/future use {33}

Not applicable, no biological specimens obtained.

## Statistical methods

### Statistical methods for primary and secondary outcomes {20a}

Repeated measure ANOVA will be used to identify group differences for ISI and the secondary outcome measures, between baseline, 12 and 24 weeks, and post hoc *t*-tests will be performed when relevant. Analyses at both timepoints are performed in order to identify an immediate and a lasting effect. Due to the novelty of the intervention, particularly in this patient group, and the rather unclear causal relationship between sleep and psychosis, we consider the secondary outcome analyses as exploratory and therefore correction for multiple testing will not be performed.

### Interim analyses {21b}

Not applicable, no interim analyses planned.

### Methods for additional analyses (e.g., subgroup analyses) {20b}

We also plan to analyze the data with a subgroup analysis of patients strictly fulfilling the TRRIP-criteria. 

### Methods in analysis to handle protocol non-adherence and any statistical methods to handle missing data {20c}

Included in the analysis population will be patients who have completed four therapy sessions. We will use the Last Observation Carried Forward (LOCF)-principle as an imputation method for handling missing data for intention-to-treat (ITT) analyses.

### Plans to give access to the full protocol, participant level-data and statistical code {31c}

The full study protocol, statistical code, and dataset will be available from the corresponding author upon request.

## Oversight and monitoring

### Composition of the coordinating center and trial steering committee {5d}

The steering committee will meet every 3rd month to evaluate the inclusion and the progression of the study.

### Composition of the data monitoring committee, its role and reporting structure {21a}

The trial is expected to be a low-risk intervention and therefore a data monitoring committee has not been considered necessary.

### Adverse event reporting and harms {22}

The symptoms of treatment-resistant patients will often fluctuate and can lead to severe events like violence or suicidal behavior. In case of worsening of psychotic symptoms or sleep disturbances, the treating psychologist will discuss with the patient whether the intervention should be terminated. The final decision to terminate is made by the psychologist.

### Frequency and plans for auditing trial conduct {23}

Not applicable, no auditing trial conduct planned.

### Plans for communicating important protocol amendments to relevant parties (e.g., trial participants, ethical committees) {25}

Important changes to the protocol will be written in amendments and in information material which will be approved by the National Ethical Committee. 

### Dissemination plans {31a}

The results of the project will be published in internationally recognized peer-reviewed journals. The project will be a Ph.D. project, and the primary author of the articles will be the Ph.D. student, JFJ. Other authorships will be allocated according to the principles of the International Committee of Medical Journal Editors (ICJME). Positive, negative, and inconclusive results will be published alike.

## Discussion

Sleep disturbances in psychiatric patients are often overlooked in clinical treatment, and with the present study, we aim at contributing to prevention, amelioration, and treatment of TRS by increasing sleep quality. TRS is a heterogenous group of patients that are heavily burdened by symptoms, suboptimal medication effect, and significant side effects. Systematically focusing on sleep to alleviate the suffering of this group is an avenue of unknown potential. If CBT-I shows positive results in TRS, it offers an easy-to-implement, cost-effective intervention.

This project involves human participants who are given one of two CBT interventions. The interventions themselves pose no real ethical issues, apart from the possible psychological discomfort associated with working on difficult subjects in therapy. The potential ethical issue of, knowingly or unknowingly, giving preferential treatment to the active treatment group is mitigated in this project by offering the active control group a CBT intervention with a specific focus on patients’ psychopathology. This is comparable in both quality and evidence base to the CBT-I intervention. This ensures that all participants in the project receive a high-quality intervention regardless of which group they are randomized into.

A core characteristic of clinical insomnia is that the affected person engages in compensatory strategies, for example, extension of total time spent in bed, withdrawal from occupational or private commitments, and/or inappropriate use of substances (illicit or otherwise) to either stay awake or fall asleep. In the CBT-I intervention, we employ SRT by restricting participants’ sleep opportunity (the time they are allowed to be in bed) for a period. We wish to inhibit the compensatory strategies that participants engage in, which perpetuate their sleep disturbances. However, we need to be careful in applying this therapeutic tool, since sleep deprivation is a factor in triggering and perpetuating psychosis. By using sleep restriction, we are, in a sense, lowering participants’ psychosis threshold, which is potentially problematic if it is not done in a controlled manner. To mitigate this risk, we have decided to not restrict below 5 h to prevent the worst sleep deprivation [42]. Furthermore, participants are seen by the therapist weekly, where they systematically assess the effect of SRT and any potential recurrence of psychotic symptoms. This enables the therapist and participant to collaborate on how to manage psychotic symptoms and when to potentially terminate the intervention. By taking these preventative steps, we want to ameliorate the potential adverse outcomes of CBT-I.

It can be challenging to adhere to a psychotherapeutic intervention, especially for people who meet the criteria for severe psychiatric disorders. Efficient psychotherapy requires sustained commitment, concentration, and a willingness to restructure thoughts and behaviors. Non-adherence is a potential pitfall, which makes the initial motivational work and sustained focus on maintaining motivation an important factor for success. During the initial contact, we will thoroughly explain the rationale and mechanisms of the interventions to enhance transparency and participants’ understanding. We have allocated a significant amount of time in the intervention manuals for motivational work, which we will tailor for each participant based on their specific situation. Throughout treatment, the therapist will routinely monitor each participant’s progress and provide support to keep motivation high. Furthermore, the procedures laid out in the intervention manuals require that time is allocated to refresh the rationale and mechanisms throughout the sessions.

## Trial status

Protocol version 1.5, 11th June 2025.

Recruitment began 28th November 2024 and will be completed approx. 31^st^ December 2026.

As per January 12, 2026, 10 patients have been screened, 5 patients have been included, and 3 patients have completed the trial.

## Data Availability

We plan to publish the final results in a peer-reviewed journal, whether they are positive, negative, or inconclusive. Furthermore, the final dataset from the study will be provided upon request.

## Abbreviations

CBT: Cognitive behavioral therapy
CBT-I: Cognitive behavioral therapy or insomnia
FAST: Functioning Assessment Short Test
GAF: Global Assessment of Functioning Scale
ISI: Insomnia Severity Index
PANSS: Positive and Negative Syndrome Scale
PSG: Polysomnography
PSP: Personal and Social Performance Scale
PSQI: Pittsburgh Sleep Quality Index
QPR: The Process of Recovery Questionnaire
SCT: Stimulus control therapy
SE: Sleep efficiency
SL: Sleep latency
SRT: Sleep restriction therapy
TIB: Time in bed
TRS: Treatment-resistant schizophrenia
TST: Total sleep time
UCS: Unit for Complex Schizophrenia
WASO: Wake after sleep onset
WHO-5: WHO Well-being Index
